# PACTUS: A two-week radiotherapy regimen for patients with anal squamous cell carcinoma unsuitable for standard chemoradiotherapy—a retrospective single-centre experience

**DOI:** 10.1016/j.ctro.2026.101280

**Published:** 2026-09-16

**Authors:** Catherine Burgos Delgado, Erik Olsson, Ola Norrlid, Nina Cavalli-Björkman, Calin Radu

**Affiliations:** Department of Immunology, Genetics and Pathology, Uppsala University, Uppsala, Sweden

**Keywords:** Anal squamous cell carcinoma, Anal cancer, Palliative radiotherapy, Hypofractionated palliative radiotherapy, Local tumor control, Symptom control, Quality of life

## Abstract

**Introduction:**

Anal squamous cell carcinoma (ASCC) is rare, and optimal treatment for patients unsuitable for definitive chemoradiotherapy remains undefined. This study describes PACTUS (Palliative Anal Cancer Treatment Uppsala Style), a two-week radiotherapy regimen designed for symptom relief and potentially durable local control.

**Methods:**

We retrospectively reviewed consecutive patients with ASCC treated with palliative-intent radiotherapy at Uppsala University Hospital (2017–2025). The principal cohort received PACTUS (40 Gy in 10 fractions). Patients treated with Short-course or Quad Shot were included as contextual case series. Treatment selection was based on patient fitness, anticipated tolerance, prognosis, logistics, and patient preference. Overall survival (OS) was measured from radiotherapy initiation. Clinical response, symptom outcomes, and adverse effects were summarized using evaluable denominators.

**Results:**

Of 735 patients treated for ASCC, 35 (4.7%) received palliative-intent radiotherapy: 28 received PACTUS, 4 Short-course, and 3 Quad Shot. The median age was 80 years (range, 60–97), 25 (71%) were women, and 30 (86%) had an ECOG performance status ≥3. Elective nodal irradiation was delivered to 24/28 patients in PACTUS. The median OS in PACTUS cohort was 30 months. At the data cut-off (April 2, 2026), 20 patients had died: 14/28 (50%) in PACTUS, 4/4 (100%) in Short-course, and 2/3 (67%) in Quad Shot. Median follow-up among surviving patients was 20 months (range, 4–71 months). Clinical response was evaluable in 15/35 (43%) patients and symptom outcomes in 13/35 (37%); therefore, these findings were not considered representative of the full cohort. Toxicity was not collected systematically or graded prospectively.

**Conclusions:**

PACTUS was feasible in this selected cohort of predominantly elderly and comorbid patients and warrants prospective evaluation. However, because treatment selection was guided by patient fitness and anticipated treatment tolerance, comparisons between the alternative regimens are descriptive only and cannot establish comparative effectiveness or a survival benefit.

## Highlights

• PACTUS is a two-week RT regimen for patients with ASCC unsuitable for standard CRT.
• PACTUS was delivered to 28 predominantly elderly and highly comorbid patients.
• The regimen aims to provide symptom relief and durable locoregional control.
• Median overall survival after PACTUS was 30 months, but treatment-selection bias limits interpretation.
• Prospective evaluation with standardized clinical and toxicity outcomes is warranted.

## Introduction

Anal squamous cell carcinoma (ASCC) [1] is a relatively rare malignancy [2], [3]. Combined chemoradiotherapy (CRT) is the standard curative treatment and has substantially improved survival outcomes [1], [4], [5]. Radiotherapy (RT) [6] alone has an important role when standard CRT is unsuitable [7], [8].

In the palliative setting, the primary goal is to relieve symptoms and improve quality of life [9]. Recommended palliative RT regimens generally use 2–5 Gy per fraction delivered over 5–15 fractions [10], [11]. However, the optimal dose-fractionation regimen for patients with ASCC unsuitable for curative-intent treatment remains undefined. Two hypofractionated palliative RT regimens are used in clinical practice: Short-course, supported by rectal-cancer data [6], [12] and Quad Shot [13], primarily studied in head and neck cancer [14], [15]. Evidence for either regimen in ASCC is limited.

Uppsala University Hospital is one of four Swedish ASCC centres, treating approximately 100 new patients annually from a large catchment area.

**PACTUS** (Palliative Anal Cancer Treatment Uppsala Style) was developed to provide a short RT regimen aiming for both local tumor control and symptom relief, including pain, bleeding, and obstructive symptoms. The regimen was designed to be completed within two weeks, preferably spanning only one weekend, to minimize time away from home. The primary objective was to describe treatment delivery, clinical outcomes, and survival following PACTUS. Short-course and Quad Shot were included only for descriptive context because treatment allocation was non-random.

## Material and methods

### Study design and population

This retrospective single-centre cohort study screened all consecutive patients treated for ASCC at Uppsala University Hospital between 2017 and 2025. The analytic cohort included patients who received RT with palliative intent. Patients without histologically confirmed ASCC were excluded.

Palliative intent was decided on when standard CRT was considered unsuitable, with the aim of symptom control, durable local control, or both. PACTUS should be regarded as an individualized, abbreviated RT regimen rather than conventional low-dose palliative RT.

For most patients, the decision not to offer standard CRT was multifactorial. Treatment allocation in all patients was initially discussed at the national multidisciplinary anal cancer conference after patient visits. Twenty-six patients were considered unsuitable due to advanced age, substantial comorbidity and poor WHO/ECOG performance status. Four patients had incurable stage IV disease with liver, lung, or peritoneum metastases. Additional reasons included another metastatic malignancy (*n* = 1) or a synchronous incurable primary malignancy (*n* = 4).

Two patients were reclassified from definitive to palliative treatment and subsequently received PACTUS. One patient with TNM stage T4N1cM0 and an ECOG performance status of 1–2 was found to have previously unrecognized peritoneal metastases on the RT planning computed tomography (CT). The second patient had a T4N1cM0 disease, an ECOG performance status of 2–3, and radiological suspicion of a synchronous lung cancer. Chemotherapy was prescribed at a 50% reduced dose because of poor clinical condition and severe cachexia (body mass index, 16 kg/m^2^; weight, 48 kg). The patient's condition deteriorated, precluding completion of definitive CRT. Treatment was discontinued after 10 fractions and reclassified as palliative.

### Treatment selection

Three schedules were used: PACTUS, 40 Gy in 10 fractions; Short-course, 25–32.5 Gy in 5 fractions; and Quad Shot, 14–15.6 Gy in 4 fractions over two consecutive days, repeated every 3–4 weeks for up to three cycles (Table 1). Treatment selection was based on clinical assessment of patient fitness, disease extent, prognosis, logistics, and patient preference. PACTUS was generally selected when a two-week regimen was considered feasible, whereas Short-course or Quad Shot was preferred for patients with limited treatment tolerance. No predefined regimen-specific criteria or validated frailty assessment were used; therefore, treatment allocation was subject to substantial confounding by indication, precluding causal comparisons between regimens.

**Table 1:** Palliative RT regimens doses and cycles CTV-T doses according to BED and EQ.D2.

REGIMENS	N/F	d (Gy)	T (d)	BED⁎ (⍺/β = 8 Gy) (Tk = 20 d)	EQ.D2⁎ (⍺/β = 8 Gy) (Tk = 20 d)	BED⁎⁎ (⍺/β = 8 Gy)	EQ.D2⁎⁎ (⍺/β = 8 Gy)
PACTUS	9	4.3	11	59.5	47.6	59.5	47.6
	10	4	12	60.0	48.0	60.0	48.0
	11	4	17	66.0	52.8	66.0	52.8
Short-course	5	5	5	40.6	32.5	40.6	32.5
	5	6.5	5	58.9	47.1	58.9	47.1
Quad Shot	4	3.5–3.9†	2	20.1–23.2	16.1–18.6	20.1–23.2	16.1–18.6

For all palliative regimens, treatment duration was shorter than the assumed kick-off time for accelerated repopulation (Tk = 20 days), resulting in identical time-corrected and non-time-corrected BED/EQ.D2 values. BED = nd[1 + d/(α/β)].

CTVT: clinical target volume; BED: biologically effective dose; EQ.D2: Equivalent dose at 2 Gy per fraction; N/F: number of fractions; d (Gy) dose per fraction in Gy, T: overall treatment time in days; Tk: Kick-off time (days until repopulation starts).

⁎ γ/α = 0,7 Gy/d, time-dependent repopulation factor per day;

⁎⁎ No time-corrected BED and EQ.D2;

† 2 fractions per day.

Patients were treated between 2017 and 2025. PACTUS and Short-course were used throughout the study period, while Quad Shot was introduced in 2022.

### Endpoints, follow up, and vital status

The principal outcome was overall survival (OS), calculated from the first day of RT to death from any cause or censoring at the date when vital status was last confirmed, which was available for all patients.

The data cut-off was April 2, 2026. Vital status was obtained from the electronic medical records, which are routinely updated with deaths recorded in the Swedish Population Register, independent of attendance at clinical follow-up. This allowed loss to clinical follow-up to be distinguished from unavailable vital status.

The first post-treatment clinical assessment was planned within two months after RT. Subsequent assessment was individualized and commonly transferred to the referring region; it could consist of clinical examination, telephone follow-up, or symptom-triggered review. Tumor response and symptom response were analyzed separately. Clinical complete response (cCR) was defined as no evidence of residual tumor on clinical examination. Partial response (PR) was defined as a reduction in tumor size or tumor-related symptoms without complete tumor disappearance. Stable disease (SD) was defined as no significant change in tumor size or symptoms, and progressive disease (PD) as tumor growth, worsening symptoms, or new disease sites during follow-up. For each outcome, results were reported both among all treated patients and among evaluable patients; missing, undocumented, death-before-assessment, and loss-to-clinical-follow-up categories were recorded separately.

### Toxicity assessment

Adverse effects were extracted retrospectively from available records. Toxicity was not collected using a predefined schedule and was not consistently graded using CTCAE (common terminology criteria for adverse events); therefore, absence of a documented event was not interpreted as absence of toxicity. Acute toxicity was defined as occurring from RT initiation through 90 days after completion, and late toxicity as occurring thereafter. Treatment interruptions, incomplete treatment, unplanned hospitalization, and deaths within 30 and 90 days were recorded as feasibility and safety outcomes.

### Radiotherapy: target delineation and delivery

Target delineation was based on institutional practice. The clinical target volume of the primary tumor (CTV-T) was generated by expanding the gross tumor volume (GTV) by 15 mm, adjusted to anatomical boundaries. The involved nodal CTV (CTV—N1) was generated by expanding the involved nodal GTV by 5 mm, adjusted to anatomical boundaries. Elective nodal irradiation (ENI, defined as CTV—N) included the nearest uninvolved nodal stations considered at risk for microscopic disease. Treatment volumes were individualized according to patient fitness and disease extent, with ENI used when durable locoregional control was intended and considered tolerable.

All patients were positioned supine for RT. Most underwent both magnetic resonance imaging and CT for treatment planning.

PACTUS was delivered using volumetric modulated arc therapy (VMAT) with a simultaneous integrated boost (SIB), predominantly using a single arc. In the standard 10-fraction schedule, the primary tumor and pathological lymph nodes ≥2 cm received 4.0 Gy per fraction, pathological lymph nodes <2 cm received 3.6 Gy per fraction, and elective target volumes received 3.2 Gy per fraction. A 7-mm planning target volume (PTV) margin was applied. Daily image guidance was performed using cone-beam CT (CBCT) combined with C-RAD surface-guided positioning. Organ-at-risk dose constraints are summarized in Table 2.

**Table 2:** Organ-at-risk (OAR) dose constraints for the 10-fraction PACTUS regimen.

OAR	Dose metric	Optimal constraint	Mandatory constraint
*Bladder*	Dmean	30 Gy	–
*Bowel bag*	D195cc	30 Gy	34 Gy
*Bowel bag*	D450cc	21 Gy	24 Gy
*Femur, left/right*	D2%	38 Gy	–
*Genitalia*	Dmean	16 Gy	–
*Testis, left/right*	Dmean	1.9 Gy	–
*Bone marrow*	Dmean	25 Gy	–

*Treatment completion:* One patient had a prolonged treatment interruption and subsequently received 11 fractions following a partial response. Schedule deviations occurred in seven additional PACTUS patients, who received 9 fractions of 4.3 Gy due to public holidays.

### Radiobiological and dose calculations

Biologically effective dose (BED) [16] was calculated using the linear-quadratic model, BED = nd[1 + d/(α/β)], with α/β = 8 Gy. Equivalent dose in 2-Gy fractions was calculated as EQ.D2 = BED/[1 + 2/(α/β)] (Table 1).

### Other anticancer treatment

In PACTUS cohort, two patients initially started definitive CRT before the treatment intent was changed to palliative RT, whereas the second received both capecitabine and mitomycin C at 50% of the standard dose. Definitive CRT was discontinued after 6 and 10 fractions, respectively. One patient with liver metastases achieved stable disease following chemotherapy and subsequently underwent PACTUS six months later.

### Statistical analysis

Categorical and continuous variables were summarized descriptively. Kaplan–Meier estimates were used to describe OS, with 95% confidence intervals and numbers at risk. Analyses were performed in GraphPad Prism version 11.0.2 for Mac.

### Ethics

The study was conducted in accordance with the General Data Protection Regulation and was approved by the Swedish Ethical Review Authority (Dnr 2022-01206-01, 2023-02327-01, and 2026-03399-02).

## Results

### Cohort

**Fig. 1:**
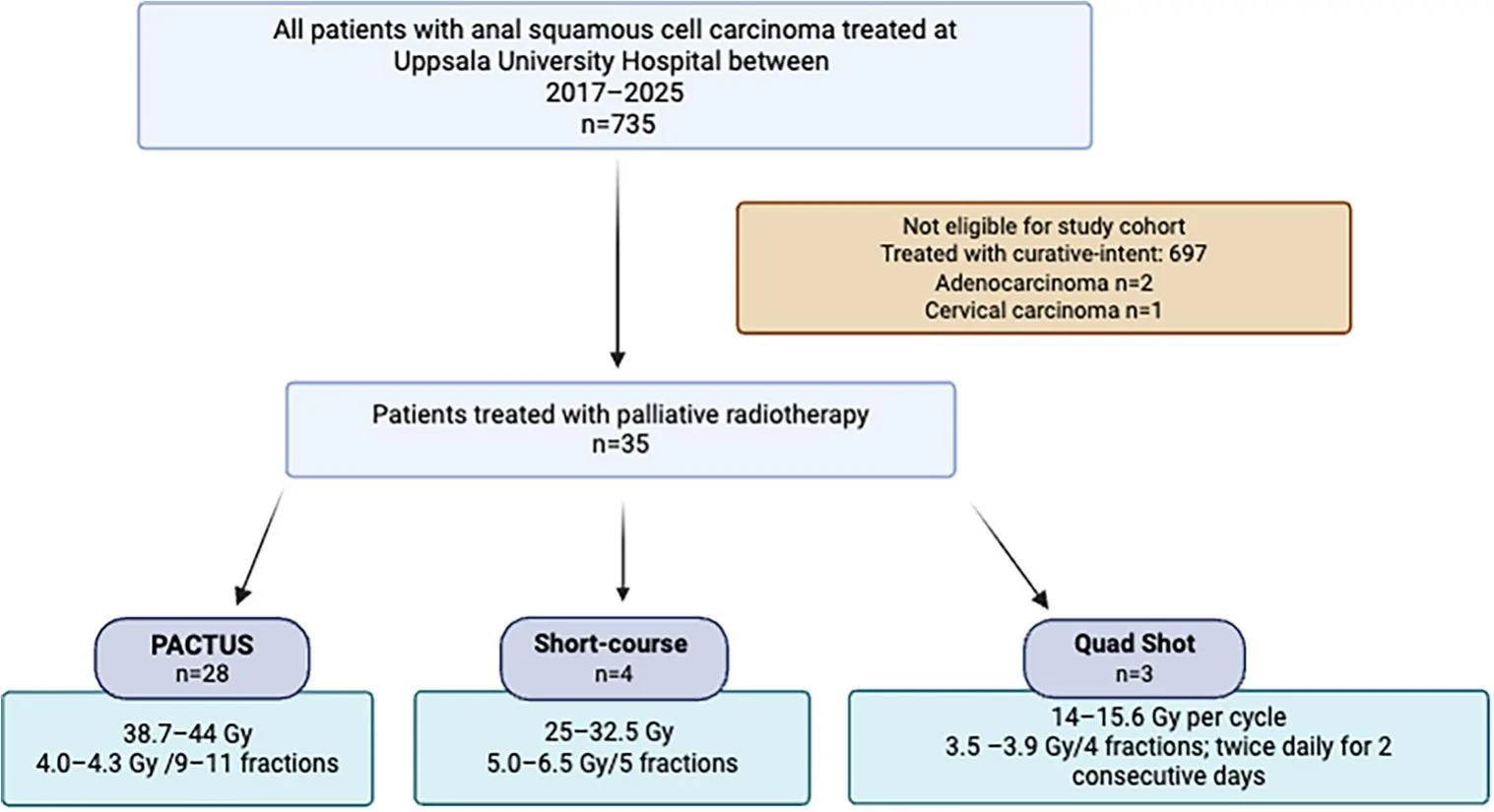
Study flow chart. 735 patients were treated for ASCC at Uppsala University Hospital between 2017 and 2015. Of these 35 patients received palliative RT treatment were included in the study cohort. Patients were treated with the PACTUS regimen (*n* = 28), Short course RT (*n* = 4) or Quad Shot (*n* = 3).

Between 2017 and 2025, 735 patients were treated for ASCC at Uppsala University Hospital. Of these, 697 received curative-intent treatment or other non-palliative treatment strategies and were excluded. A total of 38 patients received palliative-intent RT; three patients were excluded at baseline due to histopathological findings: 2 patients had adenocarcinoma and 1 had cervical carcinoma. The final cohort compromised 35 patients (4.7% of the total ASCC population): 28 (80%) patients were treated with the PACTUS, 4 (11%) received a Short-course and 3 (9%) were treated with the Quad Shot (Fig. 1).

### Patient characteristics

Median age was 80 (range 60–97) and 25 (71%) were female. Seventeen (49%) patients were former or current smokers, 26 (74%) had a p16-positive tumor, while p16 status was unavailable for 6 (17%) patients; Thirty patients (86%) had an ECOG performance status of ≥3 and 34 (97%) had a Charlson Comorbidity Index (CCI) ≥5, the prespecified definition of high comorbidity burden. Bleeding was the primary presenting symptom in 11 (31%) patients, followed by pain in 10 (29%) patients. Twenty-six (74%) patients had locally advanced disease, defined as a tumor ≥40 mm and/or node-positive disease; 19 (54%) patients had nodal involvement and 8 (23%) had metastatic disease (Table 3).

**Table 3:** Baseline patient characteristics according to palliative RT regimen.

	PACTUS *n* = 28 (80%) N (%)	Short-course *n* = 4 (11%)	Quad Shot *n* = 3 (9%)
Age (median; range)	80 (60–97)	81 (76–83)	78 (76–81)
<65 years	2 (7)	0	0
>65 years	26 (93)	4 (100)	3 (100)

Gender			
Male	9 (32)	2 (50)	0
Female	19 (68)	2 (50)	3 (100)

Smoking			
Non-smoking	12 (43)	1 (25)	1 (33)
Former/current	13 (46)	2 (50)	2 (67)
NA	3 (11)	1 (25)	0

p16 status			
positive	24 (86)	2 (50)	0
negative	3 (11)	0	0
NA	1 (3)	2 (50)	3 (100)

ECOG-Performance status			
0–1	1 (4)	0	0
2	4 (14)	0	0
3–4	23 (82)	4 (100)	3 (100)

CCI-Index			
<5 points	1 (4)	0	0
≥5 points	27 (96)	4 (100)	3 (100)

Symptoms at diagnosis			
Pain	7 (25)	3 (75)	0
Bleeding	11 (39)	0	1 (33)
Palpable mass	6 (22)	1(25)	1 (33)
Other	4 (14)	0	1 (33)

Locally advance⁎			
No	6 (22)	3 (75)	0
Yes	22 (78)	1 (25)	3 (100)

Tumor size			
<40 mm	6 (22)	3 (75)	0
≥ 40 mm	22 (78)	1 (25)	3 (100)

Nodal status			
N0	13 (46)	3 (75)	0
N+	15 (54)	1 (25)	3 (100)

Metastatic sites			
Non-metastatic	24 (86)	4 (100)	2(67)
Distant N+⁎⁎	1 (3)	0	1(33)
Visceral	3 (11)	0	0

Abbreviations: CCI: Charlson comorbidity index; NA: not available.

⁎ T3-T4N0–1 M0, T0-T4N1M0–1.

⁎⁎ Distant lymph nodes included common iliac nodes, para-aortic nodes, and lymph nodes above the diaphragm.

### Treatment delivery and volumes

Among the 28 patients treated with PACTUS, twenty received 10 fractions, seven received 9 fractions, and one received 11 fractions. Short-course consisted of 6.5 Gy × 5 fractions in three patients and 5 Gy × 5 fractions in one patient. Quad Shot was delivered as 3.5 Gy twice daily over two consecutive days for three cycles in one patient and for two cycles in another. The remaining patient received a single cycle of 3.9 Gy twice daily over two consecutive day (Table 1).

Involved nodes were included when present in all PACTUS or Short-course patients. In Quad Shot 2/3 (67%) patients received treatment targeting only the primary tumor. ENI was delivered to 24 (86%) PACTUS patients, 2 (50%) in Short-course and 1 (33%) in Quad Shot. These differences in treated volume further limit comparisons between schedules.

Hospitalization was planned for 16 /28 patients (57%) treated with PACTUS, whereas 12 (43%) were treated as outpatients. Two (7%) patients in PACTUS required unplanned hospitalization within one week of completing RT, one because of tumor bleeding and the other because of nutritional difficulties. All patients treated with Short-course (4/4) or Quad Shot (3/3) underwent planned hospitalization.

### Treatment context and change of intent

The two patients who were initially planned for curative CRT but subsequently received PACTUS had different clinical outcomes. The patient whose treatment intent changed because previously unrecognized peritoneal carcinomatosis was detected died 6 months after the start of RT. The second patient, whose treatment was reclassified as palliative because of deteriorating performance status, was alive 20 months after RT initiation at the data cut-off. The suspected synchronous lung cancer was subsequently ruled out.

Subsequent local treatment was administered to two patients: one received contact RT 22 months after PACTUS because of PD, and the other received two cycles of Quad Shot (total dose 29.6 Gy) 34 months after PACTUS, followed by contact RT 36 months after PACTUS for persistent residual tumor pain.

### Overall survival

**Fig. 2:**
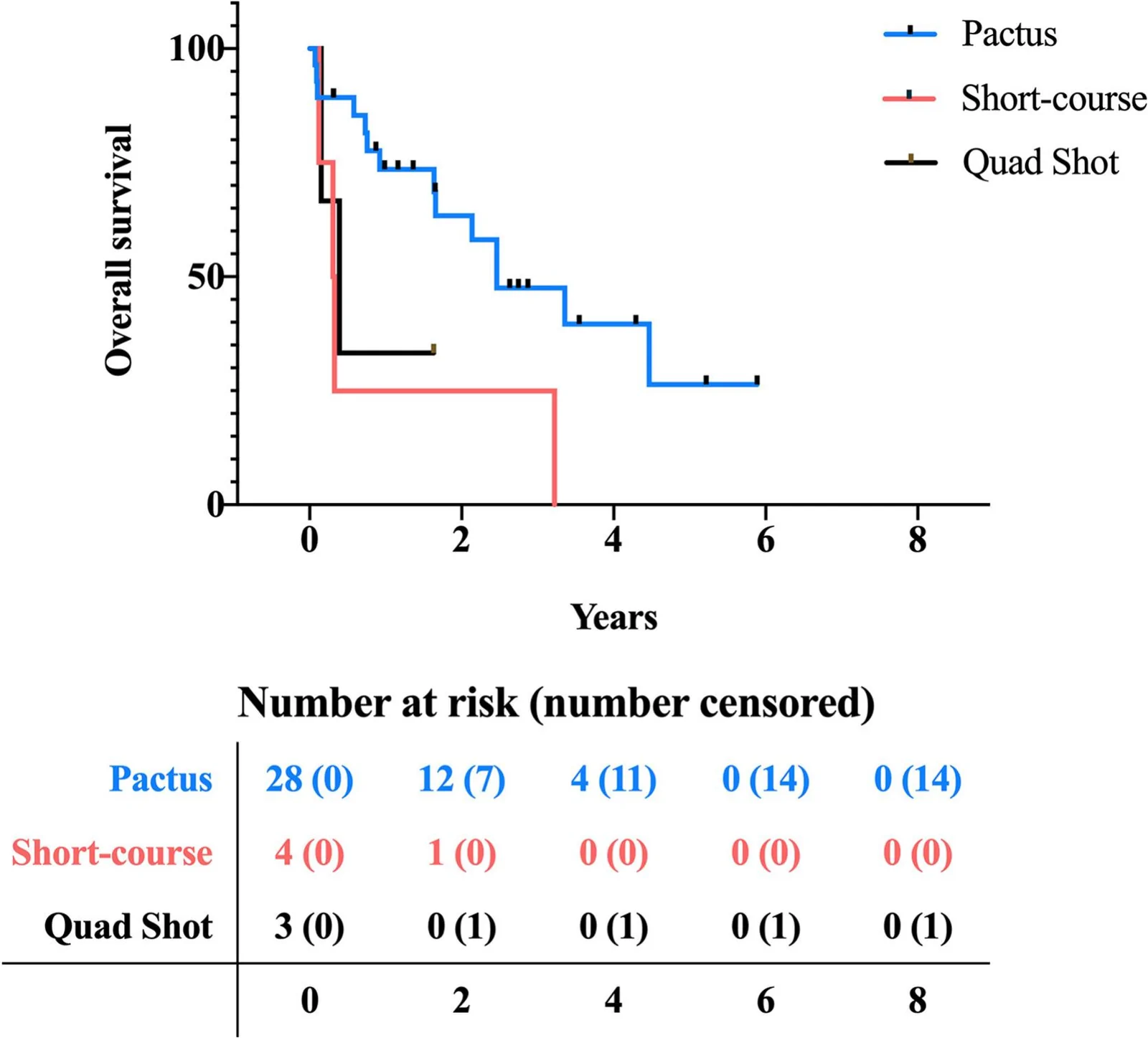
Kaplan-Meier estimates of overall survival according to palliative radiotherapy regimen.

At the data cut-off, 20/35 patients had died: 14/28 (50%) after PACTUS, 4/4 (100%) after Short-course, and 2/3 (67%) after Quad Shot. Median follow-up among surviving patients was 20 months (range, 4–71 months). Median OS was 30 months after PACTUS and 4 months in each contextual group (Fig. 2). These unadjusted estimates describe clinically selected groups and should not be interpreted as evidence that PACTUS improved survival.

The cause of death was unknown in 12/20 patients (60%). Among the 8/20 (40%) patients for whom the cause of death was known, 2 died from ASCC, 5 from causes related to multimorbidity, and 1 from complications of severe hematuria attributed to late RT toxicity following PACTUS, with no evidence of PD. Given the high proportion of unknown causes of death and the non-randomized treatment allocation, OS was used as a descriptive outcome rather than a measure of comparative RT efficacy.

Among the 2 patients whose cause of death was attributed to ASCC, one died 17 days after completing PACTUS. According to the medical record, the primary cause of death was progressive cancer rather than treatment-related toxicity. The patient was elderly, had advanced Alzheimer's disease, and required increased analgesia following RT. No other symptoms or signs suggestive of treatment-related toxicity were documented. The other patient received 2 cycles of Quad Shot and achieved a good response after the first cycle. However, at the clinical assessment before the planned 3 cycle, the patient's condition had deteriorated, and further RT was not indicated.

Among the 20 patients without a documented response assessment, 4 died before the first post-treatment clinical assessment. One was the previously described patient who died 17 days after completing PACTUS from progressive ASCC. Another was an elderly patient with substantial comorbidity who died from circulatory shock, according to the medical record. For the remaining two patients, the cause of death was not documented.

### Clinical response and symptom outcomes

Clinical response within two months was documented for 15/35 (43%) patients and was undocumented for 20/35 (57%). For patients with available follow-up data, 12/15 (80%) after PACTUS, 1/15 (7%) after Short-course and 2/15 (13%) after Quad Shot.

Among the 12 evaluable PACTUS patients, 11 (92%) achieved a cCR and 1 (8%) PR. The single evaluable Short-course patient had PD. Of the 2 evaluable Quad Shot patients, 1 achieved cCR and 1 PR. However, the high proportion of missing data prevents inference about response in the full cohort. At six months, clinical information was available for 11/35 patients (31%) and unavailable for 24/35 (69%). A persisting cCR was documented in 8 PACTUS patients and 1 Quad Shot. The evaluable Short-course patient and the other Quad Shot patient had PD.

Symptom outcomes were documented for 13/35 (37%) patients with symptom relief in 8/13 (62%): seven treated with PACTUS and one with Quad Shot, corresponding to 8/35 (23%) of the full cohort. Given the high proportion of undocumented outcomes, these findings cannot be considered representative of the full cohort.

### Adverse effects and early events

Documented adverse effects after PACTUS included faecal incontinence and mild pain. As adverse effects were neither collected systematically nor graded, this finding cannot be interpreted as a toxicity rate or evidence of treatment tolerability. Late toxicity was documented in one patient treated with PACTUS, who developed persistent severe hematuria associated with cystitis considered potentially related to RT. The patient died 12 months after treatment. The Quad Shot patient with an initial PR developed rapid progression after the second cycle and died shortly thereafter.

## Discussion

This study describes a two-week RT regimen for a rare and clinically challenging patient population, predominantly elderly and highly comorbid. The median OS of 30 months after PACTUS is noteworthy but must be interpreted cautiously, as selection based on patient fitness and prognosis resulted in substantial confounding by indication.

The designation of PACTUS as palliative requires some nuance. The regimen delivered 40 Gy in 10 fractions, and 86% of patients received ENI, with treatment goals sometimes extending beyond symptom relief to durable locoregional control. In these respects—dose intensity, elective nodal treatment, and treatment goal—PACTUS differs from Short-course and Quad Shot and represents a distinct palliative RT approach. Although both Short-course and Quad Shot have been used for palliation, these regimens are primarily symptom-directed and deliver lower cumulative doses than PACTUS [9], [13]. The reasons standard CRT was unsuitable, intended treatment goal, and use of ENI should therefore be explicit when PACTUS is considered. As OS is influenced by patient and treatment factors and disease control was not systematically assessed, it cannot be considered a surrogate for local efficacy.

Clinical response was unavailable in 57% of patients, symptom outcomes in 63%, and six-month clinical follow-up in 69%. Follow-up was mainly symptom-driven, reflecting real-world care across a large catchment area but resulting in substantial missing outcome data and potential information bias. Toxicity was not systematically assessed or graded; therefore, the few documented adverse effects cannot establish tolerability.

Additional limitations include the retrospective single-centre design, small and heterogeneous cohort, missing frailty data, and potential temporal bias. Subsequent anticancer treatments were likely few and probably had little influence on OS but the high proportion of unknown causes of death limits interpretation of cause-specific mortality. Strengths include consecutive screening of a large ASCC population, detailed characterization of a rarely studied clinical setting, and transparent reporting of missing outcome data. Prospective evaluation should standardize eligibility, treatment intent, and outcome assessment, including local control and patient-reported outcomes.

## Conclusion

PACTUS was deliverable in a selected cohort of patients with ASCC and may offer a pragmatic option when approximately two weeks of treatment and a goal of durable locoregional control are appropriate. The present data do not establish superiority over shorter schedules, and incomplete response and toxicity ascertainment precludes firm conclusions regarding efficacy or tolerability. These descriptive findings are hypothesis-generating and support prospective evaluation with standardized outcome assessment. A prospective evaluation is planned to define eligibility criteria and systematically collect patient characteristics and clinical outcomes.

## Ethics declaration

The authors have NOT obtained informed consent from participants or their legal representatives. Patient consent was not required for this study due to its retrospective design. The requirement for informed consent was specifically evaluated during the ethical review process, and the Swedish Ethical Review Authority determined that individual informed consent could be waived.

This study was performed in compliance with relevant laws, regulatory frameworks and guidelines where the research took place.

This study was approved by the Swedish Ethical Review Authority.

(Approval No. Dnr: 2022-01206-01, 2023-02327-01, and 2026-03399-02)

## Authors contribution

Conception and design: CBD, EO, CR and NC-B. Collection and assembly of data: CBD, CR, EO and ON. Data analysis and interpretation: all authors. Writing and approval of the final version of the paper: all authors.

## Declaration of competing interest

The authors declare that they have no known competing financial interests or personal relationships that could have appeared to influence the work reported in this paper.

## Data Availability

The data that support the findings of this study are available on request from the corresponding author. The data are not publicly available due to ethical restrictions protecting the privacy of the research participants.
